# CT Features of Advanced Pericochlear Otosclerosis: Case Report and a Reappraisal of Nomenclature

**DOI:** 10.2174/0115734056379053250712052649

**Published:** 2025-07-22

**Authors:** Rowa A. Mohamed, Mohamed S. Muneer, Tarik F. Massoud

**Affiliations:** 1 Saint Agnes Medical Center, Fresno, California, CA, USA; 2 Department of Radiology, Division of Neuroimaging and Neurointervention, Stanford University School of Medicine, Stanford, California, CA, USA

**Keywords:** Cochlea, Cochlear implantation, Ear, Inner hearing loss, Conductive otosclerosis, Temporal bone, Computed tomography, X-ray

## Abstract

**Background:**

This case study aimed to report the rare computed tomography (CT) features of advanced pericochlear otosclerosis, with an emphasis on a proposed new nomenclature to describe the imaging findings.

**Case Presentation:**

A 70-year-old woman with recurrent rhinosinusitis presented to our center for clinical management. The CT scan revealed the incidental rare findings of advanced retrofenestral otosclerosis in the form of extensive symmetrical pericochlear tubular lucencies in bilateral otic capsules. We coined the new term “C-hoop earring” sign for this CT appearance. She was asymptomatic and declined further audiological or imaging evaluation.

**Conclusion:**

Herein, the CT features of advanced pericochlear otosclerosis are described and the imaging and clinical connotations of the presence of the C-hoop earring sign are reviewed. This new terminology provides a more intuitive description of the imaging findings in the temporal bones for clearer understanding and communication in clinical radiological practice and education.

## 1 INTRODUCTION

Otosclerosis is a primary osteodystrophy of the otic capsule, causing pathological remodeling (spongiosis/ resorption, and sclerosis/deposition) of normally dense endochondral bone [1-3]. It is frequently responsible for acquired conductive, sensorineural, or mixed hearing loss in adults [4]. In its advanced stages, pericochlear otosclerosis manifests on CT as an area of demineralization coursing around the membranous cochlea [5], to produce a so-called “double ring” or “fourth ring of Valvassori” imaging sign, first introduced in 1985 [6, 7]. However, we believe this term to be a longstanding misnomer because neither does the abnormal perichochlear bony lucency form a complete ring, nor is there a second ring for it to be labelled as a double ring. Instead, we coin the new, descriptive, and more accurate term, “C-hoop earring” sign, for these appearances of advanced perichochlear otosclerosis on CT. This chronic stage of disease may be asymptomatic in about half of the patients [5], and therefore has a likelihood of being detected incidentally on head or dedicated temporal bone imaging for other indications. We submit that this new terminology provides a more intuitive, easier to remember, and more accurate description of the imaging findings. There is less ambiguity than referring to “two rings”, and a C-hoop earring can be easily remembered in the context of a pathology related to the otic capsule and inner ear. We believe this would also enhance clarity in communication regarding this condition in clinical neuroradiological practice and resident education.

## 2 CASE REPORT

**Fig. (1):**
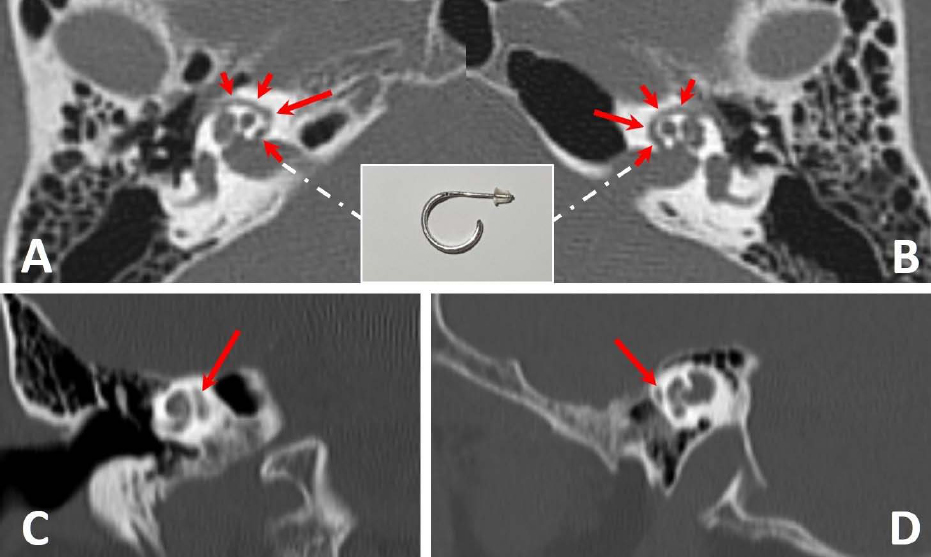
A 70-year-old woman with incidental advanced pericochlear otosclerosis showing the C-hoop earring sign. Sinus CT images on bone algorithm and windows show (**A**) right, and (**B**) left magnified axial images of the temporal bones at mid-cochlea levels with bilateral, symmetric, well-marginated, curvilinear tubular-like (1.1 mm in diameter) lucencies (red arrows) within the otic capsules, extending on each side from the middle ear cavity just anterior to the oval window anterolaterally, to the internal auditory canal posteromedially, and around the cochlea, consistent with advanced pericochlear (retro-fenestral) otosclerosis. No otospongiotic changes are present. The inset image shows the analogous C-hoop earring. (**C**) and (**D**) show magnified coronal and sagittal images of the right and left temporal bones, respectively, at mid-cochlea levels with lucencies (red arrows) representing slices through the “C-hoop earring” of pericochlear otosclerosis.

A 70-year-old woman with paranasal sinus symptoms had a non-contrast sinus CT performed using a standard protocol on a 64-slice helical CT scanner (LightSpeed; GE Medical Systems, Waukesha, WI). Axial images were obtained with 120 kVp, 280 mA (Smart mA adjusted), rotation time = 0.7 s, pitch = 0.75, field of view = 20 cm, and matrix = 512 × 512 using a bone algorithm and producing contiguous 0.625 mm slices for orthogonal reconstruction, also including dorsally extended views of the temporal bones. Multiplanar reformatted images on bone windows showed the incidental rare findings of advanced bilateral, symmetric pericochlear otosclerosis (Fig. **1**) for description). She was asymptomatic and declined further audiological or imaging evaluation. A paranasal sinus CT performed 23 years previously had shown similar but unreported indolent temporal bone findings.

## 3 DISCUSSION

Otosclerosis was first described by Antonio Maria Valsalva in 1735 when he referred to stapedial fixation as a cause of hearing loss [8]. Politzer (1893) later described pathological changes in the otic capsule [8], which consist of disordered bone resorption, new bone formation, and vascular/connective tissue stroma proliferation [5]. It first develops in the third and fourth decades of life, occurs more than twice in females than in males [9], and is bilateral in 80% of individuals [4]. Although idiopathic, it is considered an autosomal-dominant hereditary disease with variable penetrance and possible links to persistent viral infection or autoimmunity [10]. TGFβ-1 protein implicated in bony remodeling may play a key role in the pathogenesis of otosclerosis [11]. Recently, several strong associations have also been established between new potential candidate genes and otosclerosis [12]. Most cases of otosclerosis are asymptomatic, especially when bilateral [9]. When symptomatic, otosclerosis is one of the commonest causes (18–22%) of acquired progressive conductive hearing loss [9], with a prevalence of 0.3–0.4% in the general population [13].

High-resolution, thin-section, multiplanar reformatted CT is the imaging technique of choice for the evaluation of otosclerosis changes in the petrous temporal bones because it can show very subtle bone findings as it has a >90% sensitivity for otosclerosis detection [9]. Recent photon-counting and ultra-high resolution CT of the temporal bones allow for improved spatial resolution of 0.12 mm [14]. Small otosclerotic lesions can sometimes be challenging to visualize on CT scans. In that regard, a recent study by Emin *et al*. showed that the diagnostic performance of an artificial intelligence algorithm was comparable to that of a trained radiologist, although sensitivity at an estimated ideal threshold was lower than for radiologists [15]. Of note, neuroradiologists reporting on high-resolution CT scans of the temporal bones have high overall rates for the detection of otosclerosis at over 82% compared to much lower rates in less experienced general radiologists [16]. MRI has been used for otosclerosis evaluation [17, 18], and this technique can also demonstrate the C-hoop earring sign [19]; however, CT remains the gold standard

The use of preoperative imaging evaluation for suspected otosclerosis is well-established. As outlined by Mangia *et al*. [2], CT evaluation is especially recommended in the following instances: (1) in cases of isolated sensorineural or mixed hearing loss when an otosclerosis diagnosis may be unclear clinically [4]; (2) in children, particularly in boys to rule out X-linked mixed hearing loss; (3) in the presence of facial deformities or ear malformations; (4) with intermittent hearing loss; (5) with associated vestibular impairment; (6) for revision cases; (7) in cases with a history of trauma; and (8) with a history of chronic otitis media. The CT findings dictate the type of treatment for hearing loss [1]. Elective stapedoplasty is the treatment for antefenestral involvement, and hearing aid treatment is preferred to cochlear implant for retrofenestral disease owing to difficult electrode insertion caused by the cochlear osteodystrophy that increases the risk of complications [1].

Many CT grading systems for otosclerosis have been proposed based on location and severity of imaging appearances, but there is no universally accepted system [2, 4]. These classification systems are clinically relevant because, when otosclerosis is graded as severe, there is also an increased severity of sensorineural hearing loss and a higher risk of facial nerve stimulation after cochlear implantation [4]. Two common classification systems are those of Rotteveel and Symons and Fanning [2, 4]. Both systems describe low grades for fenestral involvement and higher grades for varying retrofenestral bony changes around the cochlea or diffusely throughout the otic capsule. According to both classifications, advanced pericochlear otosclerosis can result in a “double ring effect” (corresponding to grade/type 2c otosclerosis [2]), owing to the confluence of spongiotic foci surrounding the cochlea within the thickness of the otic capsule [4]. We coined an alternative term (C-hoop earring sign) for this CT appearance, and although many patients with chronic otosclerosis are asymptomatic, we acknowledge that the lack of hearing assessment in our reported case limits the depth of understanding of this disease vis-à-vis this C-hoop earring sign.

Histology, spatial location, and temporal factors can all contribute to explaining the origin and CT appearance of this “C-hoop earring” sign. Histologically, otospongiosis results in circumscribed foci of new, soft, vascular bone, instead of the normally avascular bone found in adults [9]. Purported cartilaginous remnants trapped within endochondral bone of the otic capsule undergo remodeling and are replaced by highly vascular, immature bone (as summarized by Çanakcı H and Bülbül [9]). The resorption of perivascular bone in this spongiotic phase produces radiolucent foci that are commonly found in three locations [5]: anterior to the oval window (antefenestral) at the fissula in 96.1% of otosclerotic temporal bones, anterior part of the internal auditory canal (IAC) in 46.8%, and the pericochlear region in 26%. Notably, perichoclear spongiosis appears to never occur alone, but may occur with concurrent antefenestral spongiosis (in 5.2%), and may be confluent with both anterior-IAC and antefenestral spongiosis (in 20.8%) [5] to form the C-hoop earring appearance. It has been observed that approximately 10% of petrous temporal bones with fenestral otosclerosis develop long-term retrofenestral involvement [4]. Yagi *et al*. suggest that the C-hoop earring appearance initially starts from antefenestral, followed by anterior-IAC. Lastly, these two locations become confluent with perichoclear spongiosis in the final stage [5]. Indolent progression toward the C-hoop earring appearance likely varies between individuals, but there is no apparent correlation between the extent of disease and age or hearing level [5]. In our patient, similar but unreported indolent temporal bone findings were observed 23 years earlier, thus attesting to the indolence of this phase of disease. Long-term follow-up of patients with the C-hoop earring sign may be necessary in more patients to determine the dynamic evolution of this imaging sign.

## CONCLUSION

It is unclear why the less-than-accurate term “double ring sign” was coined in the first instance some four decades ago [6]. We propose that this nomenclature be upgraded using the “C-hoop earring sign” as a more apt description of the CT features of advanced pericochlear otosclerosis. We understand that the adoption of new terminology requires consensus, and we hope this proposal will stimulate constructive discussion toward that goal. We trust that the proposed new term for this imaging sign will eventually achieve broad recognition and see wider usage in both the literature and future clinical studies.

## Data Availability

Not applicable.

## ABBREVIATION

CT: Computed tomography

## STANDARDS OF REPORTING

CARE guidelines were followed.
